# Hydrogen Sulfide Modulates Microglial Polarization and Remodels the Injury Microenvironment to Promote Functional Recovery After Spinal Cord Injury

**DOI:** 10.1111/cns.70431

**Published:** 2025-05-14

**Authors:** Yu Wang, Xinyi Jia, Yuqi Zhang, Haibin Shi, Yuhui Sun, Yaobo Liu

**Affiliations:** ^1^ Jiangsu Key Laboratory of Drug Discovery and Translational Research for Brain Diseases, Institute of Neuroscience, Soochow University, Departments of Rehabilitation Medicine and Neurology, The Fourth Affiliated Hospital of Soochow University Suzhou China; ^2^ State Key Laboratory of Radiation Medicine and Protection, School of Radiation Medicine and Protection, and Collaborative Innovation Center of Radiological Medicine of Jiangsu Higher Education Institutions Soochow University Suzhou China; ^3^ Co‐Innovation Center of Neuroregeneration, Nantong University Nantong China

**Keywords:** functional recovery, hydrogen sulfide, neuroinflammation, scar formation, spinal cord injury

## Abstract

**Aims:**

Spinal cord injury (SCI) disrupts tissue homeostasis, leading to persistent neuroinflammation and scar formation that severely impedes functional recovery. Current therapeutic approaches are insufficient to address these challenges. In this study, we investigated whether exogenous hydrogen sulfide (H_2_S) can modulate neuroinflammatory responses and remodel the injury microenvironment to promote tissue repair and restore motor function following SCI.

**Methods:**

T10 crush SCI was induced in mice, followed by daily intraperitoneal administration of the H_2_S donor anethole trithione (ADT). Immunofluorescence staining, tissue clearing, western blotting, and behavioral assessments were performed to evaluate scar formation, vascular regeneration, neuronal survival, and motor function.

**Results:**

ADT‐based H_2_S therapy significantly promoted wound healing, inhibited scar formation, enhanced vascular regeneration, and protected residual neurons and axons from secondary injury. Mechanistically, H_2_S suppressed microglial proliferation and activation, promoting their polarization toward an anti‐inflammatory phenotype and alleviating neuroinflammation. Consequently, motor function recovery was markedly improved.

**Conclusion:**

H_2_S modulates microglial activation and mitigates neuroinflammation, establishing a permissive microenvironment for SCI repair and significantly enhancing motor function recovery. Given ADT's established clinical safety and its effective gasotransmitter properties, our findings underscore its immediate translational potential for treating SCI.

## Graphical abstract

Hydrogen sulfide remodels the injury microenvironment by inhibiting scar formation, enhancing vascular regeneration and neuronal survival, and reducing neuroinflammation, ultimately improving motor functional recovery after spinal cord injury.

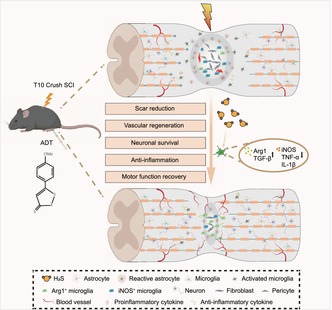

## 1 Introduction

Spinal cord injury (SCI) is a common traumatic disorder typically caused by external impacts on the spine, leading to vertebral fractures or dislocations that compress or transect the spinal cord. This results in partial or complete loss of sensory and motor functions below the level of injury [1, 2, 3]. The pathophysiological process of SCI extends beyond the initial mechanical injury and involves a complex cascade of secondary injury events. Secondary injury comprises various cellular and molecular mechanisms, including vascular damage, cell death, inflammatory responses, axonal dieback, and scar formation [3, 4]. Following SCI, the microenvironment undergoes profound changes, encompassing diverse cell types and their secreted factors [5, 6]. The injury microenvironment plays a crucial role in the repair process, as it can either promote tissue regeneration or hinder recovery [7].

Following SCI, cells within the damaged area release various pro‐inflammatory factors, such as tumor necrosis factor (TNF‐α) and interleukins (IL‐1β, IL‐6), which trigger a local inflammatory response and recruit immune cells, including microglia and macrophages. While inflammation facilitates the clearance of cellular debris and prevents infection, prolonged inflammation creates a microenvironment that is detrimental to neuronal survival and axonal regeneration [8, 9]. Additionally, a dense scar forms at the injury site, comprising a fibrotic core surrounded by a glial scar [10]. This scar, a critical component of the injury microenvironment, has a dual role in SCI repair. On one hand, scar formation supports tissue self‐repair; on the other, excessive scar accumulation forms a physical and molecular barrier that obstructs neural regeneration [11, 12, 13, 14]. Thus, modulating the inflammatory response and remodeling the injury microenvironment represent promising strategies for promoting neural regeneration and functional recovery.

In recent years, the role of gaseous signaling molecules, such as hydrogen sulfide (H_2_S), nitric oxide (NO), and carbon monoxide (CO), in the central nervous system (CNS) has gained considerable attention [15, 16, 17]. As endogenous signaling mediators, these gaseous molecules perform diverse biological functions, including regulating inflammation, mitigating oxidative stress, and enhancing cell survival [18, 19]. Among them, H_2_S has demonstrated neuroprotective effects in several CNS diseases, such as Alzheimer's disease, Parkinson's disease, and traumatic brain injury [20, 21, 22, 23, 24]. Research indicates that H_2_S plays a crucial role in maintaining blood–brain barrier integrity, protecting neurons, promoting myelin regeneration and axonal repair, and preserving mitochondrial function [25, 26]. The role of H_2_S in SCI has also garnered significant research interest [27]. Studies reveal that the H_2_S donor NaHS can reduce blood‐spinal cord barrier permeability by inhibiting endoplasmic reticulum stress and autophagy, thereby facilitating SCI recovery [28]. Moreover, the H_2_S donor GYY4137 has been shown to reduce neuronal loss and alleviate motor dysfunction following ischemia‐reperfusion [29]. Despite growing evidence supporting the therapeutic potential of H_2_S in SCI, the mechanisms by which it modulates the injury microenvironment remain unclear.

ADT [5‐(4‐methoxyphenyl)‐3H‐1,2‐dithiole‐3‐thione] is a widely used slow‐release H_2_S donor with notable advantages over other H_2_S donors due to its stable and gradual release profile, which enhances its efficacy for prolonged therapeutic applications [30, 31]. Studies have shown that ADT effectively protects neurons from glutamate‐induced oxidative toxicity by gradually releasing H_2_S, thereby minimizing oxidative damage and neuronal apoptosis [30]. Additionally, in a cerebral ischemia model, ADT demonstrated anti‐inflammatory and neuroprotective effects by inhibiting neuroinflammation, reducing infarct size, and improving neurological outcomes [32]. These findings support ADT's neuroprotective potential as a promising candidate for SCI treatment. This study aims to investigate the regulatory effects of H_2_S on the SCI microenvironment through intraperitoneal administration of ADT. The findings will elucidate novel therapeutic targets for H_2_S in SCI and pave the way for developing innovative strategies to enhance SCI recovery.

## 2 Materials and Methods

### 2.1 Animals

Wild‐type C57BL/6J mice were purchased from Shanghai SLAC Laboratory Animal Co. Ltd. (Shanghai, China). Aldh1l1‐P2A‐iCre mice with a C57BL/6JGpt genetic background and Rosa26(R26)‐CAG‐LSL‐tdTomato(TdT) mice with a B6J;B6N genetic background were obtained from GemPharmatech (Nanjing, China). These strains were crossed to generate the Aldh1l1^iCre^::R26‐TdT transgenic mice. In this model, the Aldh1l1 promoter drives the specific expression of iCre in astrocytes, thereby activating TdT for precise astrocyte labeling. All animals used in the study were adult males, 8–10 weeks of age, with body weights between 20 and 25 g. Animals were housed under standard laboratory conditions, including a 12 h light/dark cycle, an ambient temperature of 22°C ± 2°C, and a relative humidity of 40%–60%. Food and water were provided ad libitum. All animal procedures were performed in accordance with the Guide for the Care and Use of Laboratory Animals and were approved by the Animal Care and Use Committee of Soochow University.

### 2.2 SCI Model and Treatment

Mice were anesthetized with 2% (w/v) Avertin, followed by a T10 laminectomy to expose the spinal cord. A complete spinal cord crush injury was induced at the T10 level using No. 5 Dumont forceps with a compression duration of 2 s. In the sham group, only a T10 laminectomy was performed without subsequent SCI to ensure the same level of exposure as the injury group. After surgery, the muscle and skin layers were sutured, and mice were placed on a warming pad to recover fully. Postoperatively, the bladder was manually expressed twice daily to facilitate urination until euthanasia.

Mice were randomly assigned to treatment groups receiving daily intraperitoneal injections of ADT (10, 20, or 40 mg/kg) or a vehicle solution (10% dimethyl sulfoxide in corn oil). Treatments began 1 day post‐SCI and continued daily until euthanasia.

### 2.3 Quantification of H_2_S in Injured Mouse Spinal Cord

To verify that the H_2_S released by ADT reaches and exerts its effects at the SCI site, we employed H_2_S‐activatable nanoprobes (ZNNPs) to directly detect the H_2_S signal at the lesion site. At 2, 8, and 24 h following the initial ADT administration, the laminectomy site was re‐exposed to reveal the lesion area, and the ZNNPs (1 mg/mL, 50 μL) were carefully applied to the SCI site. After a 30 min incubation period, fluorescence images were captured using an IVIS imaging system with an excitation wavelength of 640 nm and an emission wavelength of 720 nm [33].

### 2.4 Behavioral Analysis

#### 2.4.1 Basso Mouse Scale (BMS) Behavioral Analysis

Hindlimb locomotor recovery was assessed using the BMS in an open‐field test at 1, 3, and 7 days post‐SCI, and subsequently on a weekly basis until day 56. A detailed description of the BMS methodology can be found in the literature [34]. Two independent researchers, blinded to the treatment groups, performed the BMS scoring.

#### 2.4.2 Motion Trajectory Analysis

Prior to testing, small adhesive markers were affixed to the hip, knee, ankle, and foot joints of the mouse's right hindlimb. Digital imaging was used to capture the movement of the right hindlimb during locomotion, and the motion trajectory was reconstructed from the recorded data.

#### 2.4.3 Electromyography (EMG) Recordings

EMG recordings were performed 28 days post‐SCI. A recording electrode was implanted in the tibialis anterior (TA) muscle of the right hindlimb and connected to an EMG recording module (BIOPAC, USA) via the Biopac MP150 data acquisition system [35]. The TA muscle myoelectric burst was detected while the mice moved on a body weight‐supporting treadmill (90% support, 2 m·min^−1^; SANS, China).

### 2.5 Tissue Clearing Technique

Tissues were cleared using the CUBIC (Clear, Unobstructed Brain Imaging Cocktails and Computational Analysis) method, following previously described protocols and using specific clearing reagents to enhance transparency [36, 37]. Mice were perfused transcardially with phosphate‐buffered saline (PBS), followed by 4% paraformaldehyde, and tissues were post‐fixed overnight in 4% paraformaldehyde. Prior to clearing, the tissues were thoroughly washed with PBS.

For spinal cord clearing, tissues were immersed in 15 g of reagent‐1, prepared with 12.5 g urea (Sigma, Germany), 12.5 g N,N,N′,N′‐tetramethylethylenediamine (Sigma, Germany), 17.5 mL distilled water, and 7.5 g Triton X‐100 (MasterTech, USA), at 37°C under gentle agitation for 2 days. The solution was then refreshed, and the sample was immersed in a fresh volume of reagent‐1 for an additional 2 days. Following clearing, the spinal cord tissues were rinsed multiple times with PBS at room temperature under gentle agitation, followed by immersion in 20% (w/v) sucrose in PBS. The samples were degassed and immersed in 15 g of reagent‐2, consisting of 25 g sucrose (Sinopharm, China), 12.5 g urea, 7.5 mL distilled water, 5 g 2,2′,2″‐nitrilotriethanol (Aladdin, China), and 400 μL of 10% Triton X‐100, for 1–2 days.

Cleared spinal cord tissues were imaged using a confocal microscope (LSM700; Zeiss, Germany). Three‐dimensional (3D) reconstructions of the spinal cord and lesion sites were generated using the Surface tool in Imaris.

### 2.6 Immunohistochemistry

Mice were perfused transcardially with PBS, followed by 4% paraformaldehyde, and tissues were post‐fixed overnight in 4% paraformaldehyde. The tissues were then washed with PBS under gentle agitation and sequentially immersed in 20% and 30% (w/v) sucrose solutions. Parasagittal spinal cord sections were prepared for immunostaining using a cryostat. Primary antibodies were diluted in PBS containing 1% bovine serum albumin (BSA) and incubated with tissue sections at 4°C overnight at the following concentrations: Mouse anti‐GFAP‐Cy3 (1:500, Sigma‐Aldrich, C9205), rabbit anti‐PDGFR‐β (1:500, Abcam, ab32570), rabbit anti‐fibronectin (1:200, Abcam, ab2413), goat anti‐CD31 (1:500, R&D Systems, AF3628), rabbit anti‐NeuN (1:500, Abcam, ab177487), rabbit anti‐NF (1:200, Abcam, ab207176), rabbit anti‐Iba1 (1:500, Wako, 019‐19741), rat anti‐Ki67 (1:500, invitrogen, 14‐5698‐82), rat anti‐CD68 (1:500, Bio‐Rad, MCA1957), rabbit anti‐iNOS (1:200, Abcam, ab15323), and rabbit anti‐Arg1 (1:500, GeneTex, GTX109242). After incubation with primary antibodies, sections were washed in PBS and incubated overnight at 4°C with Alexa Fluor 488‐ or 647‐conjugated secondary antibodies (1:500, Abcam) diluted in 1% BSA/PBS. Sections were washed again with PBS prior to imaging. Confocal imaging was performed using a Zeiss LSM700 microscope, and image processing and exporting were completed with Zen software (Zeiss, Germany).

### 2.7 Western Blotting

Protein extracts were isolated from 2‐mm spinal cord tissue segments centered on the lesion site 7 days post‐SCI. Western blotting was performed according to established protocols, including SDS‐PAGE, electroblotting, and enhanced chemiluminescence (ECL) detection. Primary antibodies were used at a dilution of 1:1000 and included the following: Rabbit anti‐PDGFR‐β (Abcam, ab32570), rabbit anti‐fibronectin (Abcam, ab2413), rabbit anti‐TGF‐β (Abclonal, A2124), rabbit anti‐TNF‐α (Abclonal, A20851), rabbit anti‐IL‐1β (Abclonal, A20527), rabbit anti‐Arg1 (GeneTex, GTX109242), and rabbit anti‐iNOS (Abcam, ab15323). Secondary antibodies were used at a dilution of 1:5000 and included rabbit anti‐HRP (Fdbio, FDG007). Rabbit anti‐β‐tubulin (1:5000, Fdbio, FD0064) was used as the internal loading control. Immunoreactive bands were semi‐quantitatively analyzed using ImageJ software. Analysis included background subtraction and normalization to the loading vehicle group.

### 2.8 Statistical Analysis

All statistical analyses were performed using GraphPad Prism (Version 8.0.1). Data are presented as mean ± SEM. Normality was confirmed using the Shapiro–Wilk test. Comparisons between two groups were conducted with Student's *t*–test, whereas multiple group comparisons were analyzed using one‐way or two‐way analysis of variance (ANOVA). Statistical significance was defined as *p* < 0.05 for all analyses.

## 3 Results

### 3.1 Detection of H_2_S at the Injury Site in the Mouse Spinal Cord

**FIGURE 1:**
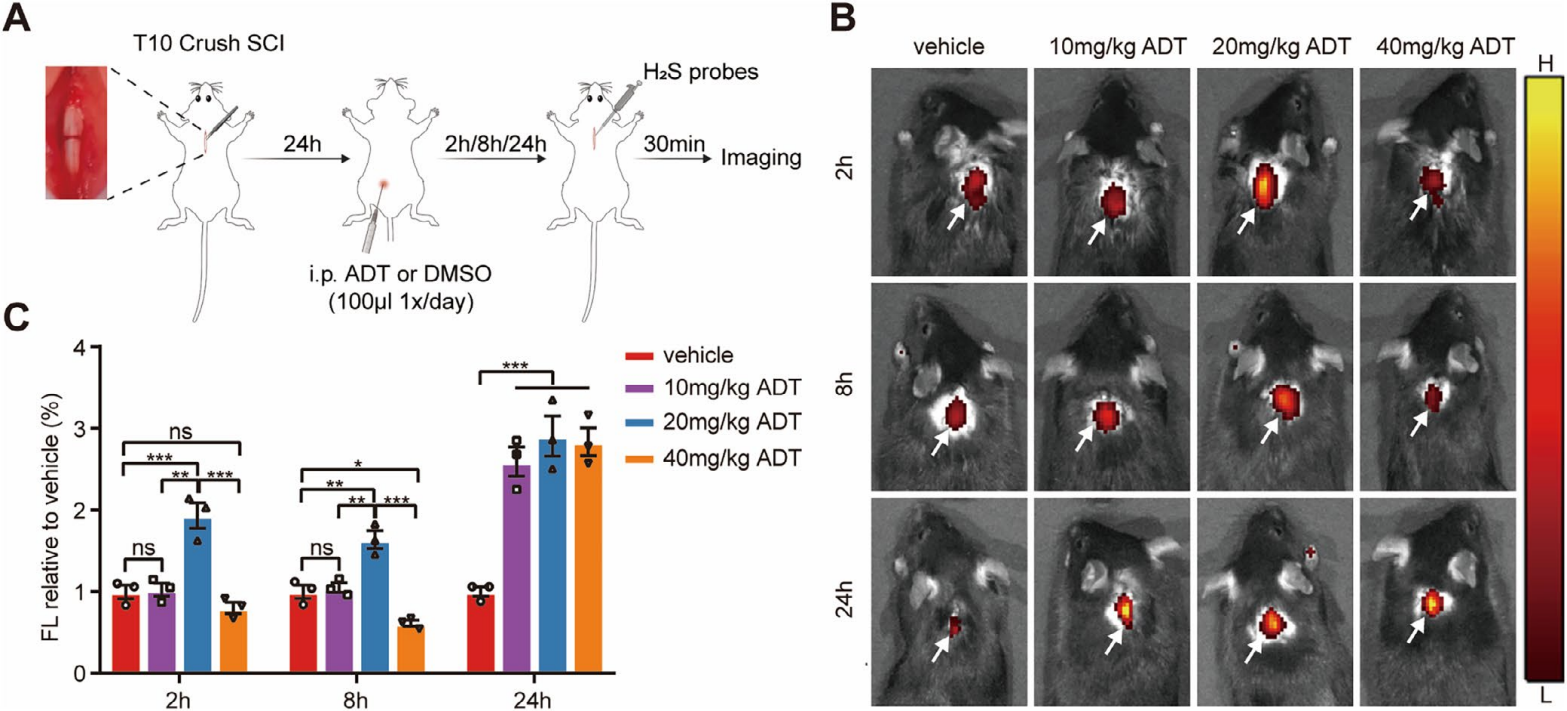
In vivo fluorescence imaging of H_2_S at the SCI site in mice after ADT administration. (A) Experimental design showing daily intraperitoneal injection of ADT (10, 20, or 40 mg/kg, 100 μL) or vehicle solution (10% dimethyl sulfoxide in corn oil, 100 μL) starting on day 1 post‐SCI until euthanasia, along with the imaging time points. (B) Representative fluorescence images showing H_2_S signals at the SCI site at 2, 8, and 24 h after ADT administration in the vehicle and ADT groups (10, 20, and 40 mg/kg). Arrows indicate the precise location of the H_2_S signals at the SCI site. (C) Quantitative analysis of H_2_S fluorescence intensity at the SCI site in the vehicle and ADT groups (10, 20, and 40 mg/kg). Data are presented as mean ± SEM; *n* = 3 per group. Statistical analysis was performed using one‐way ANOVA, followed by Tukey's post hoc test; *p < 0.05, **p < 0.01, ***p < 0.001, and ns indicates no statistically significant difference.

Mice were administered daily intraperitoneal injections of either the H_2_S donor ADT or vehicle (10% dimethyl sulfoxide in corn oil) beginning on day 1 post‐SCI and continuing until euthanasia to ensure sustained H_2_S delivery and therapeutic efficacy. In vivo fluorescence imaging was used to visualize H_2_S signals at the injury site. Based on previous research [38], we selected ADT doses of 10 mg/kg, 20 mg/kg, and 40 mg/kg to identify the optimal concentration for therapeutic efficacy. Research has shown that ADT releases H_2_S with an in vivo half‐life of approximately 8–24 h [30]. Accordingly, we evaluated H_2_S release at different time points (2, 8, and 24 h post‐administration) following the first ADT administration (Figure 1A).

Representative fluorescence images (Figure 1B) taken at various time points after administration revealed distinct H_2_S signals at the injury sites across the different ADT dosage groups. At 2 h post‐administration, the 20 mg/kg ADT group exhibited the highest fluorescence signal, whereas the 10 mg/kg and 40 mg/kg groups did not show significant differences compared to the vehicle group. At 8 h, the 20 mg/kg group still displayed the strongest signal, while the 10 mg/kg group remained similar to the vehicle, and the 40 mg/kg group exhibited a lower signal than the vehicle group. At 24 h, fluorescence signals in all ADT‐treated groups were significantly higher than those in the vehicle group, indicating a marked increase in H_2_S levels at the injury site due to ADT treatment (Figure 1C).

In summary, these findings indicate that intraperitoneal injection of ADT facilitates the sustained release of H_2_S and targeted delivery to the SCI site. Among the tested doses, 20 mg/kg demonstrated the most effective H_2_S release and distribution, exhibiting the highest fluorescence signal at early time points (2 and 8 h) and maintaining superior signal levels at 24 h, which highlight its potential therapeutic advantages.

### 3.2 H_2_S Promoted Wound Healing and Inhibited Scar Formation After SCI

**FIGURE 2:**
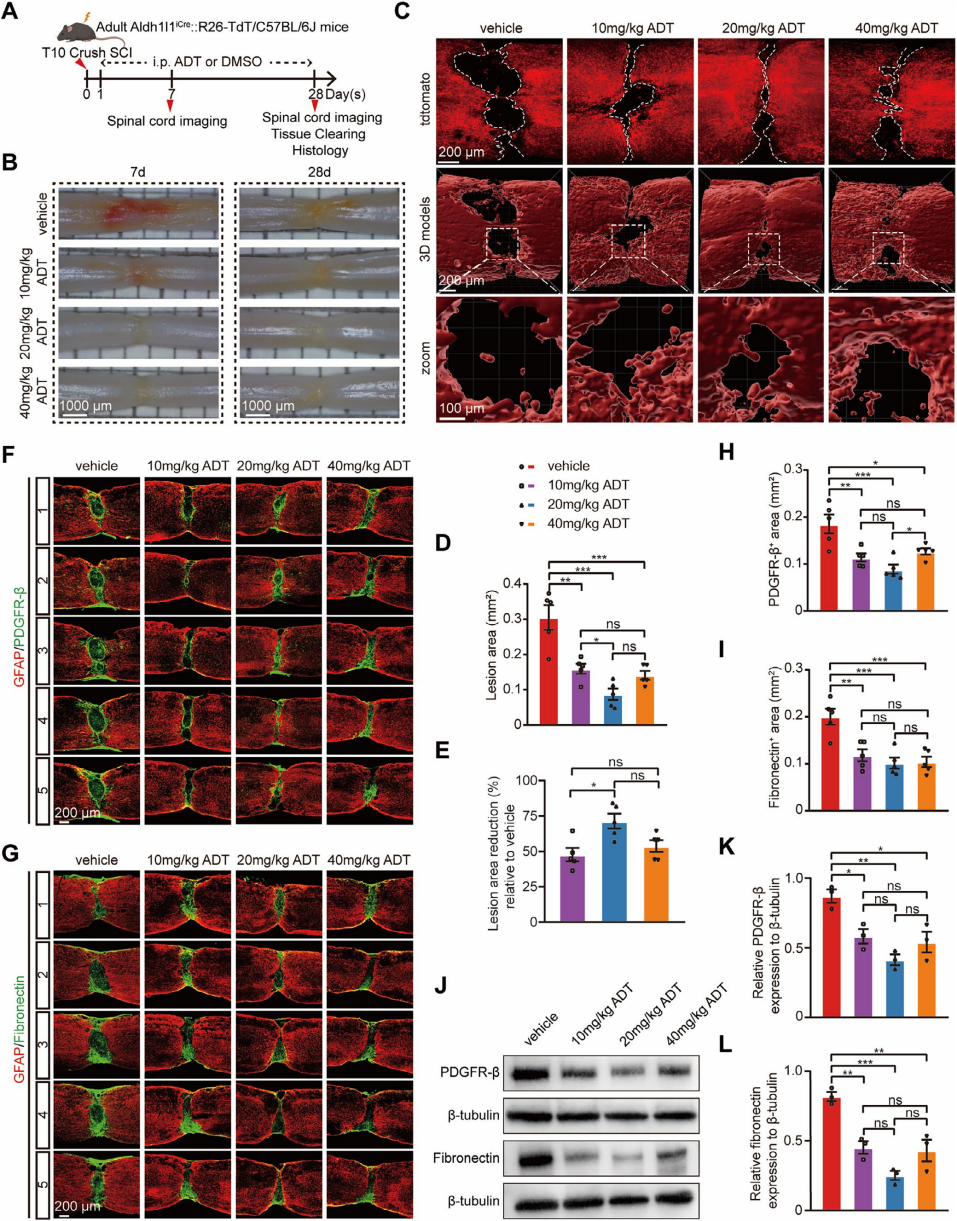
H_2_S promotes wound healing and reduces scar formation after SCI. (A) Schematic representation of the experimental design. (B) Representative high‐resolution digital surgical microscopy images of the SCI site in the vehicle, 10 mg/kg, 20 mg/kg, and 40 mg/kg ADT groups, at 7 and 28 days post‐SCI. (C) Representative immunofluorescence images and 3D reconstructions of the lesion area in the vehicle, 10 mg/kg, 20 mg/kg, and 40 mg/kg ADT groups 28 days post‐SCI. (D) Quantification of the lesion area in the vehicle, 10 mg/kg, 20 mg/kg, and 40 mg/kg ADT groups. (E) Percentage reduction in lesion area in the 10, 20, and 40 mg/kg groups relative to the vehicle group. (F, G) Representative images of five parasagittal spinal cord sections centered on the midline, stained with GFAP (red) and either PDGFR‐β (green) or fibronectin (green), in the vehicle, 10 mg/kg, 20 mg/kg, and 40 mg/kg ADT groups 28 days post‐SCI. (H) Quantitative analysis of the PDGFR‐β^+^ area in the vehicle, 10 mg/kg, 20 mg/kg, and 40 mg/kg ADT groups. (I) Quantitative analysis of the fibronectin^+^ area in the vehicle, 10 mg/kg, 20 mg/kg, and 40 mg/kg ADT groups. (J) Western blot analysis of PDGFR‐β and fibronectin expression at 28 days post‐SCI. (K, L) Quantitative analyses of the expression levels of PDGFR‐β (K) and fibronectin (L). Data are presented as mean ± SEM; *n* = 5 per group (D, E, H, I); *n* = 3 per group (K, L). Statistical analysis was performed using one‐way ANOVA, followed by Tukey's post hoc test; *p < 0.05, **p < 0.01, ***p < 0.001, and ns indicates no statistically significant difference.

We next evaluated the effects of H_2_S on wound healing and scar formation following SCI. Spinal cord tissue imaging was performed on days 7 and 28 post‐SCI to evaluate wound healing, while assessments of scar formation were conducted on day 28 (Figure 2A). Lesioned spinal cords were initially visualized using high‐resolution digital surgical microscopy. The imaging results revealed that the ADT‐treated groups displayed significantly better tissue closure and enhanced surface tissue continuity compared to the vehicle group (Figure 2B). Wound healing was further assessed using the CUBIC spinal cord clearing method in combination with Aldh1l1^iCre^::R26‐TdT transgenic mice, which label the majority of astrocytes, enabling precise 3D reconstruction of the lesion area at 28 days post‐SCI. In the vehicle group, a pronounced cavity was observed at the lesion site, whereas ADT treatment markedly reduced the lesion area (Figure 2C). Compared with the vehicle group, lesion area was reduced by 47.64% (*p* < 0.01), 71.33% (*p* < 0.001), and 53.79% (*p* < 0.001) in the 10, 20, and 40 mg/kg groups, respectively (Figure 2D). Notably, the 20 mg/kg group exhibited the most pronounced reduction. A comparative analysis of the lesion area reduction percentages relative to the vehicle group revealed that the 20 mg/kg group exhibited a reduction that was 23.69% greater than that in the 10 mg/kg group and 20.39% greater than that in the 40 mg/kg group (Figure 2E). These findings suggest that the 20 mg/kg dose, which showed the greatest reduction with significant differences, is likely to represent the optimal dose for treatment.

Scar formation involves a complex interplay of various cell types, including pericytes and fibroblasts, which contribute to the production and organization of extracellular matrix components. To assess scar formation, we performed co‐immunostaining for GFAP and either PDGFR‐β or fibronectin at 28 days post‐SCI (Figure 2F,G). The results demonstrated a marked reduction in the lesion area following ADT treatment, consistent with the 3D reconstruction findings (Figures 2C,D,F,G and S1). Quantification of pericyte‐ and fibroblast‐containing regions was carried out across five consecutive sagittal sections aligned at the midline. Our results demonstrated that the PDGFR‐β^+^ area was significantly reduced in all ADT‐treated groups compared to the vehicle group. Specifically, the reductions in PDGFR‐β^+^ area for the 10 mg/kg, 20 mg/kg, and 40 mg/kg ADT‐treated groups were 38.65% (*p* < 0.01), 52.11% (*p* < 0.001), and 31.72% (*p* < 0.05), respectively. Furthermore, the 20 mg/kg group exhibited the greatest reduction, with decreases that were 13.46% greater than that in the 10 mg/kg group and 20.39% greater than that in the 40 mg/kg group (Figure 2H). Similarly, compared to the vehicle group, the fibronectin^+^ areas in the 10 mg/kg, 20 mg/kg, and 40 mg/kg ADT‐treated groups were reduced by 40.82% (*p* < 0.01), 49.03% (*p* < 0.001), and 47.96% (*p* < 0.001), respectively. The 20 mg/kg group exhibited the greatest reduction, with decreases that were 8.20% greater than that in the 10 mg/kg group and 1.06% greater than that in the 40 mg/kg group (Figure 2I). Additionally, western blot analysis further assessed the expression of PDGFR‐β and fibronectin in the lesion area at 28 days post‐SCI (Figure 2J). The expression levels of both PDGFR‐β and fibronectin were significantly reduced in the 10 mg/kg, 20 mg/kg, and 40 mg/kg ADT‐treated groups compared to the vehicle group (*p* < 0.05, *p* < 0.01, and *p* < 0.05, respectively, for PDGFR‐β in Figure 2K; *p* < 0.01, *p* < 0.001, and *p* < 0.01, respectively, for fibronectin in Figure 2L). These findings further confirm the inhibitory effects of H_2_S on scar formation.

These findings suggest that H_2_S promotes wound healing and inhibits scar formation after SCI. Moreover, the overall therapeutic trend indicates that the 20 mg/kg ADT dose affords the most effective repair.

### 3.3 H_2_S Promoted Vascular Regeneration and Neuronal Survival After SCI

**FIGURE 3:**
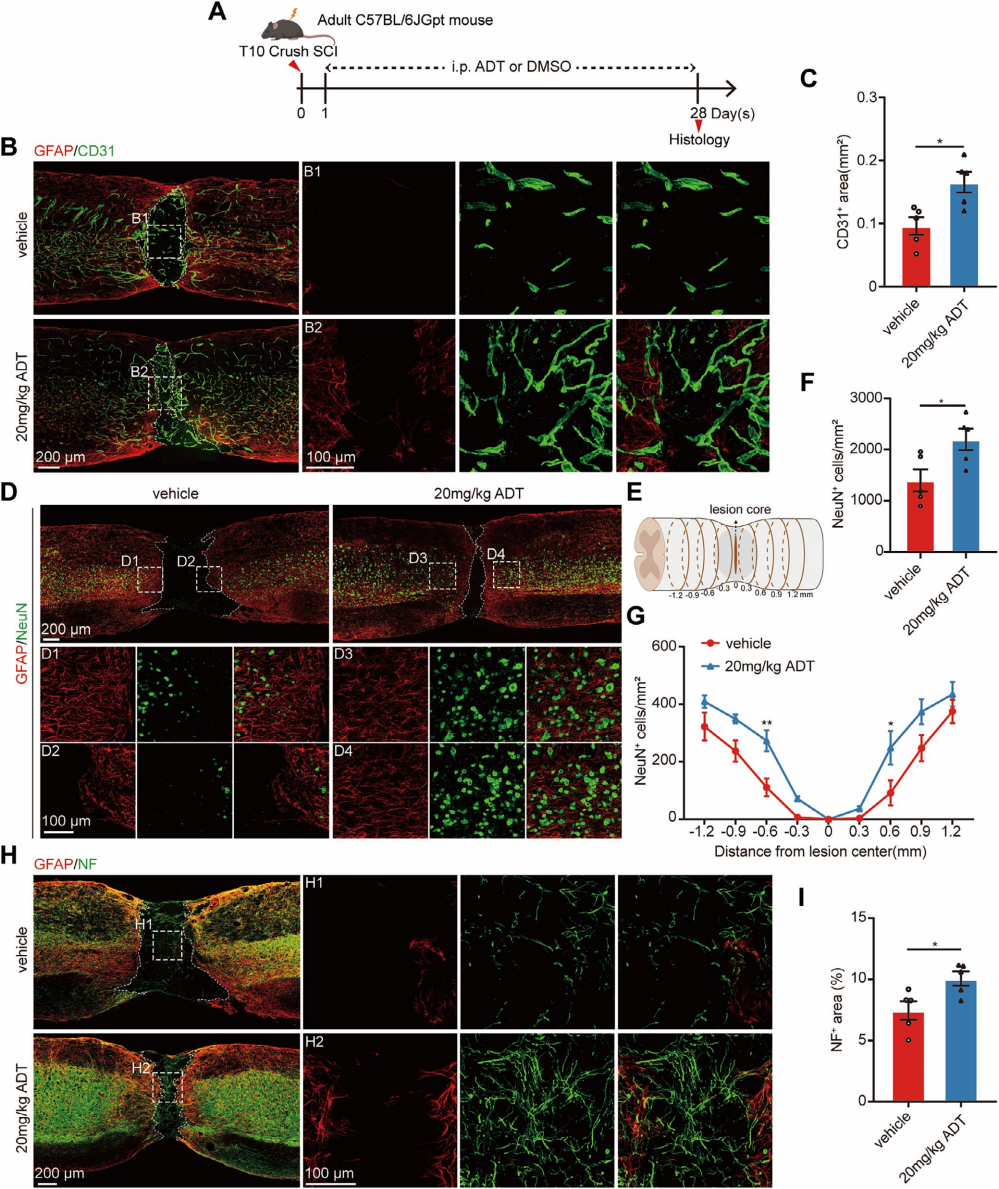
H_2_S enhances vascular regeneration and protects neurons and axons 28 days post‐SCI. (A) Schematic representation of the experimental design. (B) Representative immunofluorescence images of spinal cord sagittal sections co‐stained with GFAP (red) and CD31 (green) in the vehicle and 20 mg/kg ADT groups. B1 and B2 show higher‐magnification images of the boxed areas in (B). (C) Quantitative analysis of the CD31^+^ area at the lesion site. (D) Representative immunofluorescence images of spinal cord sagittal sections co‐stained with GFAP (red) and NeuN (green) in the vehicle and 20 mg/kg ADT groups. D1–D4 show higher‐magnification images of the boxed areas in (D). (E) Schematic diagram of the spinal cord depicting the lesion core and eight adjacent zones (at the lesion site) used for NeuN^+^ neuron quantification post‐SCI. (F) Quantitative analysis of the total number of NeuN^+^ neurons. (G) Quantitative analysis of NeuN^+^ neurons in zones surrounding the lesion boundary. (H) Representative immunofluorescence images of spinal cord sagittal sections co‐stained with GFAP (red) and NF (green) in the vehicle and 20 mg/kg ADT groups. H1 and H2 show higher‐magnification images of the boxed areas in (H). (I) Quantitative analysis of the NF^+^ area at the lesion site. Data are presented as mean ± SEM; *n* = 5 per group. Statistical analyses included an unpaired two‐tailed Student's *t*‐test for (C), (F), and (I), and a two‐way ANOVA, followed by Bonferroni's post hoc test for multiple comparisons for (G). *p < 0.05, **p < 0.01.

After SCI, microvascular structures are disrupted by inflammatory cell infiltration, resulting in secondary injury, neuronal death, and impaired axonal regeneration [1, 3]. Vascular regeneration is critical for SCI repair, as it supplies nutrients to neurons, thereby promoting neuronal survival and axonal regeneration [39]. Previous studies have demonstrated that H_2_S promotes angiogenesis and protects neural cells [40, 41, 42, 43]. Building on our previous findings that H_2_S significantly reduced tissue damage, we administered a 20 mg/kg dose of ADT to further investigate its potential effects on vascular regeneration and neuroprotection following SCI (Figure 3A).

CD31 is a well‐established endothelial cell marker used to assess vascular regeneration [44]. Immunofluorescence analysis of spinal cord sections revealed a significant increase in the CD31^+^ area at the lesion site 28 days post‐SCI in the 20 mg/kg ADT group compared to the vehicle group (Figure 3B,C). These results suggest that H_2_S treatment significantly enhanced vessel density in the 20 mg/kg ADT group, indicating its substantial impact on vascular regeneration following SCI. To evaluate neuronal survival, we stained the neuronal marker NeuN and counted neurons within regions at various distances from the lesion center (Figure 3D,E). The results revealed that a greater number of neurons survived in the 20 mg/kg ADT group (Figure 3F), with a significantly higher neuron density observed in the 300–600 μm range from the lesion center compared to the vehicle group (Figure 3G). These findings suggest that H_2_S effectively protected the neurons around the lesion site. Then, we stained the axonal fibers with anti‐NF in the spinal sections (Figure 3H). We found that more NF^+^ axons at the lesion site were present in the 20 mg/kg ADT group compared to the vehicle group (Figure 3I). Overall, these results suggest that H_2_S improves the microenvironment following SCI, thereby promoting vascular regeneration and protecting neurons and axons.

### 3.4 H_2_S Inhibited Microglial Proliferation and Accumulation After SCI

**FIGURE 4:**
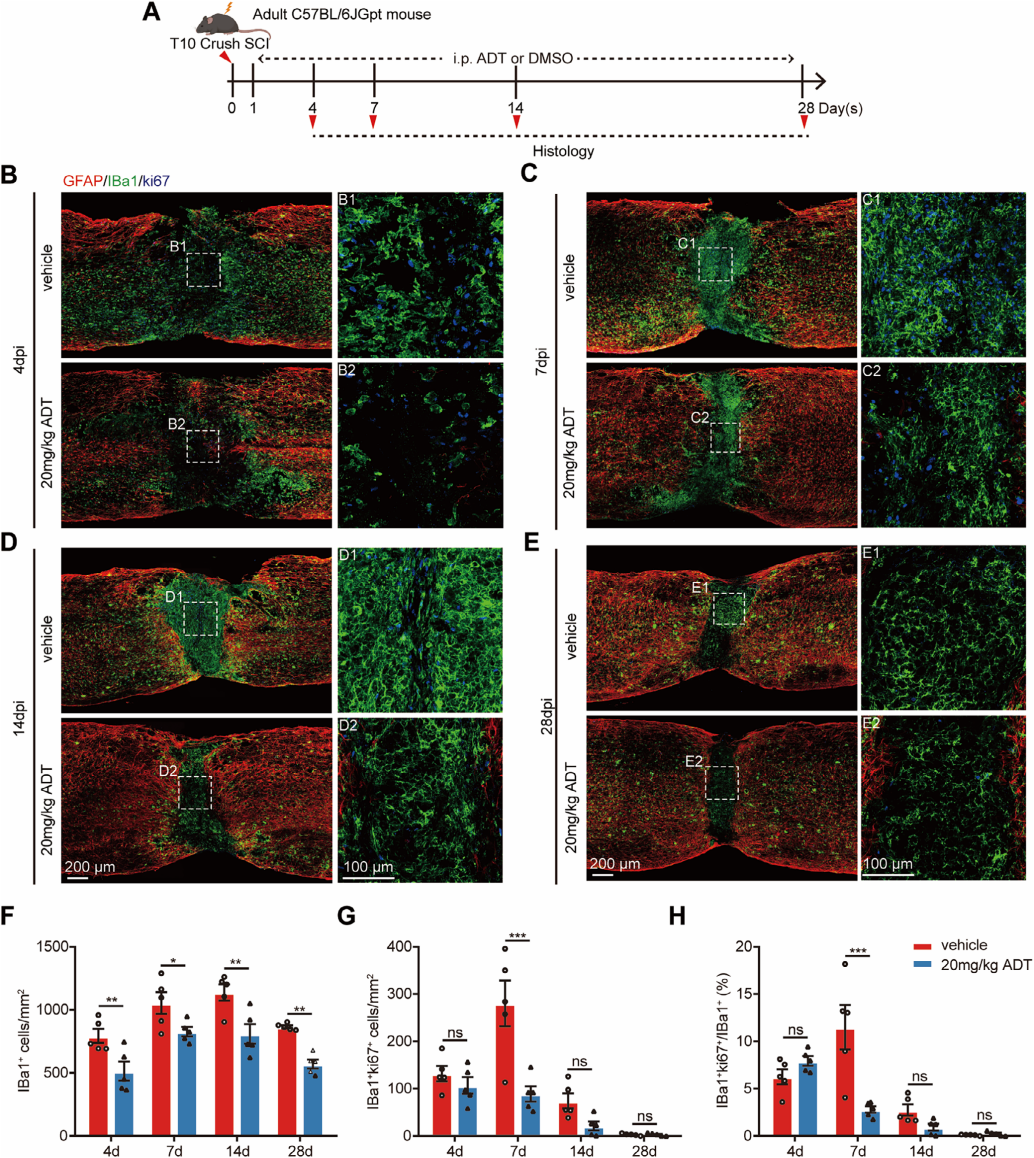
H_2_S reduces microglial accumulation at the lesion site after SCI. (A) Schematic representation of the experimental design. (B–E) Representative immunofluorescence images of spinal cord sagittal sections co‐stained for GFAP (red), Iba1 (green), and Ki67 (blue) in the vehicle and 20 mg/kg ADT groups 4, 7, 14, and 28 days post‐SCI. B1, B2, C1, C2, D1, D2, E1, and E2 show higher‐magnification images of boxed areas in (B–E). (F) Quantitative analysis of Iba1^+^ microglia in the vehicle and 20 mg/kg ADT groups at 4, 7, 14, and 28 days post‐SCI. (G) Quantitative analysis of Iba1^+^Ki67^+^ microglia in the vehicle and 20 mg/kg ADT groups at 4, 7, 14, and 28 days post‐SCI. (H) Percentage of Iba1^+^ microglia undergoing proliferation in the vehicle and 20 mg/kg ADT groups at 4, 7, 14, and 28 days post‐SCI. Data are presented as mean ± SEM; *n* = 5 per group. Statistical analyses were performed using two‐way ANOVA, followed by Bonferroni's post hoc test for multiple comparisons. *p < 0.05, **p < 0.01, ***p < 0.001, and ns indicates no statistically significant difference.

Microglia, the primary innate immune cells in the (CNS), play a pivotal role in the inflammatory response following SCI [45]. To further investigate the effects of H_2_S, we analyzed the dynamic changes in microglia following SCI. Immunofluorescence staining for the microglial marker Iba1 and the cell proliferation marker Ki67 was performed at 4, 7, 14, and 28 days post‐SCI (Figure 4A–E). The results revealed that 20 mg/kg ADT treatment significantly reduced the total number of Iba1^+^ microglial cells at multiple time points post‐SCI (Figure 4F), indicating that H_2_S effectively suppressed microglial accumulation and reduced the overall number of microglia in the lesion area.

Additionally, Iba1^+^Ki67^+^ double‐positive microglial cells, representing proliferating microglia, were analyzed. Consistent with previous studies showing that microglial proliferation peaks at 7 days post‐SCI [46], our findings revealed that H_2_S significantly inhibited microglial proliferation during this peak period (Figure 4G,H). These results suggest that H_2_S reduces the number of microglia in the lesion area by suppressing their proliferation during the critical phase, thereby mitigating excessive inflammatory responses and protecting neural tissue.

### 3.5 H_2_S Promoted Anti‐Inflammatory Microglial Polarization and Alleviated Neuroinflammation After SCI

**FIGURE 5:**
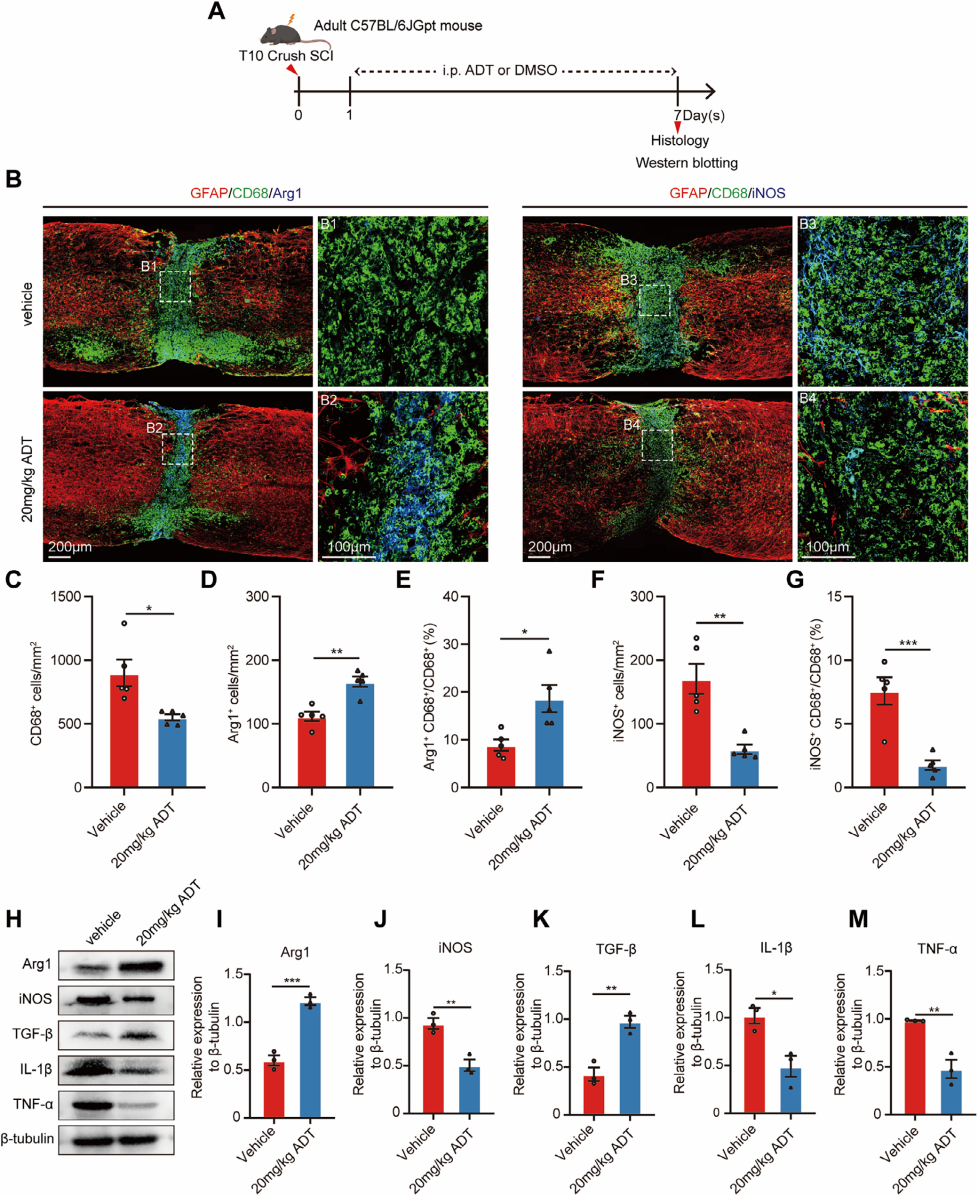
H_2_S alleviates neuroinflammation after SCI. (A) Schematic representation of the experimental design. (B) Representative immunofluorescence images of spinal cord sagittal sections co‐stained for GFAP (red), CD68 (green), and Arg1 (blue) or iNOS (blue) in the vehicle and 20 mg/kg ADT groups7 days post‐SCI. B1, B2, B3, and B4 show higher‐magnification images of boxed areas in (B). (C) Quantification of CD68^+^ microglia in the vehicle and 20 mg/kg ADT groups at 7 days post‐SCI. (D) Quantification of Arg1^+^ microglia in the vehicle and 20 mg/kg ADT groups at 7 days post‐SCI. (E) Quantification of the ratio of Arg1^+^CD68^+^ to CD68^+^ microglia in the vehicle and 20 mg/kg ADT groups at 7 days post‐SCI. (F) Quantification of iNOS^+^ microglia in the vehicle and 20 mg/kg ADT groups at 7 days post‐SCI. (G) Quantification of the ratio of iNOS^+^CD68^+^ to CD68^+^ microglia in the vehicle and 20 mg/kg ADT groups at 7 days post‐SCI. (H) Western blot analysis of Arg1, iNOS, TGF‐β, IL‐1β, and TNF‐α expression at the lesion site 7 days post‐SCI. (I–M) Quantitative analyses of the expression levels of Arg1 (I), iNOS (J), TGF‐β (K), IL‐1β (L), and TNF‐α (M) at the lesion site. Data are presented as mean ± SEM; *n* = 5 per group (C–G); *n* = 3 per group (I–M). Statistical analyses were performed using an unpaired two‐tailed Student's *t* test. *p < 0.05, **p < 0.01, ***p < 0.001.

To further investigate the effect of H_2_S on microglia and its potential to alleviate neuroinflammation following SCI, we assessed microglial activation and polarization at 7 days post‐SCI (Figure 5A). Given the crucial role of anti‐inflammatory microglia in tissue repair [47], we sought to determine whether H_2_S facilitates microglial polarization toward an anti‐inflammatory phenotype. To this end, we performed double‐labeling of the activated microglia marker CD68 alongside either the anti‐inflammatory marker Arg1 or the pro‐inflammatory marker iNOS (Figure 5B). At 7 days post‐SCI, the 20 mg/kg ADT group exhibited significantly fewer CD68^+^ activated microglia compared to the vehicle group (Figure 5C). Moreover, the 20 mg/kg ADT group displayed a higher number of Arg1^+^ microglia and a lower number of iNOS^+^ microglia relative to the vehicle group (Figure 5D,F). We further quantified the proportion of CD68^+^ microglia co‐expressing Arg1 or iNOS. In the 20 mg/kg ADT group, Arg1^+^CD68^+^ microglia accounted for 18.64% of the total CD68^+^ microglia at the lesion site, representing a 9.75% increase compared to the vehicle group (Figure 5E). Conversely, iNOS ^+^CD68^+^ microglia accounted for only 1.77% of the total CD68^+^ microglia, reflecting a 5.83% reduction compared to the vehicle group (Figure 5G). These findings demonstrate that H_2_S significantly promotes the polarization of microglia toward an anti‐inflammatory phenotype.

Western blotting analysis further confirmed that, at the lesion site on day 7 post‐SCI, the expression of Arg1 was significantly upregulated, while the expression of iNOS was downregulated in the 20 mg/kg ADT group (Figure 5H–J). To further evaluate whether the 20 mg/kg ADT treatment alleviates the neuroinflammatory environment induced by SCI, we analyzed the expression of inflammation‐associated proteins in lesioned spinal tissues via Western blotting (Figure 5H). The results demonstrated that, in the 20 mg/kg ADT‐treated group, the levels of the pro‐inflammatory markers IL‐1β and TNF‐α were significantly reduced (Figure 5L,M), while the level of the anti‐inflammatory marker TGF‐β was notably increased (Figure 5K). These findings suggest that H_2_S mitigates neuroinflammation and improves the inflammatory microenvironment post‐SCI by promoting the phenotypic transition of microglia and reducing the release of pro‐inflammatory factors.

### 3.6 H_2_S Improved Motor Function After SCI

**FIGURE 6:**
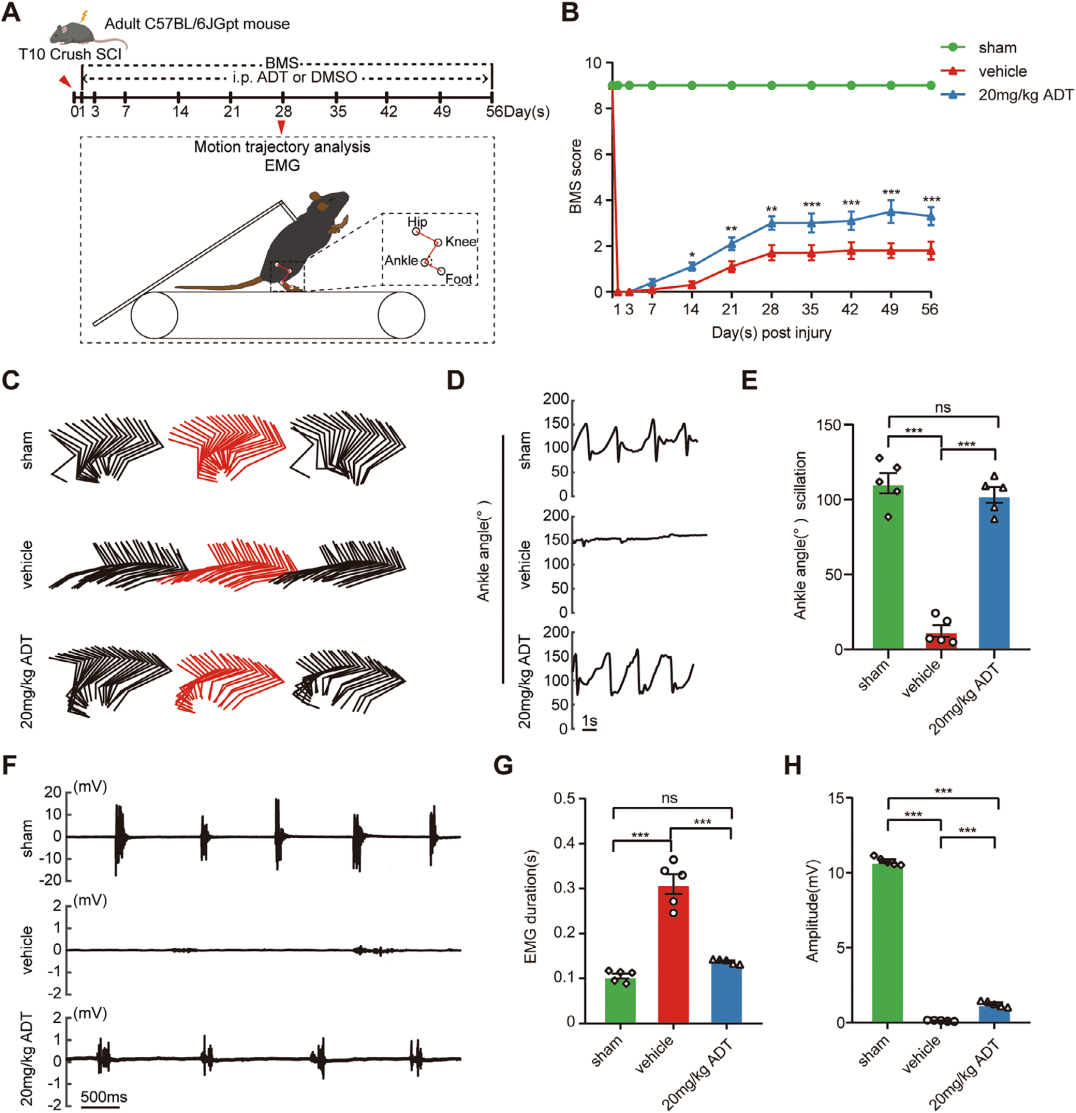
H_2_S improves motor function in mice after SCI. (A) Schematic representation of the experimental design. (B) BMS scores of mice at the indicated times post‐SCI. (C) Example stick diagrams of hindlimb movements in the sham, vehicle, and 20 mg/kg ADT groups. (D) Ankle angle degree curves in the sham, vehicle, and 20 mg/kg ADT groups. (E) Quantification of ankle angles in the sham, vehicle and 20 mg/kg ADT groups. (F) EMGs of the TA muscles in the sham, vehicle, and 20 mg/kg ADT groups. (G) Quantification of the average duration of a single TA burst in EMG in the sham, vehicle and 20 mg/kg ADT groups. (H) Quantification of the maximum amplitude of EMG bursts in the sham, vehicle and 20 mg/kg ADT groups. Data are presented as mean ± SEM; *n* = 10 per group (B); *n* = 5 per group (E, G, H). Statistical analyses were performed using one‐way ANOVA, followed by Tukey's post hoc test (E, G, H) or two‐way ANOVA, followed by Bonferroni's post hoc test for multiple comparisons (B). *p < 0.05, **p < 0.01, ***p < 0.001, and ns indicates no statistically significant difference.

Our study revealed that H_2_S exerts neuroprotective effects by modulating microglial activity, thereby improving the injury microenvironment and promoting tissue repair following SCI. To further evaluate whether H_2_S facilitates motor function recovery, we performed comprehensive behavioral assessments at 28 days post‐SCI, including BMS scoring, motion trajectory analysis, and EMG recordings (Figure 6A).

Motor function was assessed through BMS scoring at multiple time points, including days 0, 1, 3, 7, 14, 21, and up to day 56 post‐SCI. The results showed significantly higher BMS scores in the 20 mg/kg ADT group compared to the vehicle group, indicating that H_2_S positively influences motor function recovery (Figure 6B). Motion trajectory analysis revealed that, compared to sham‐operated mice, the vehicle group exhibited minimal autonomous hindlimb movement and limited ankle joint activity. In contrast, mice treated with 20 mg/kg ADT demonstrated substantial autonomous hindlimb movement, with ankle joint flexion closely resembling that of the sham group (Figure 6C–E). Additionally, EMG activity of the right hindlimb TA muscle was analyzed at 28 days post‐SCI (Figure 6F). The results indicated that the 20 mg/kg ADT group exhibited a significant increase in EMG burst amplitude and a marked reduction in the duration of individual EMG signals compared to the vehicle group (Figure 6G,H).

Importantly, Hematoxylin and eosin (HE) staining of the heart, liver, spleen, lung, and kidney confirmed that a 20 mg/kg ADT intraperitoneal injection did not induce significant long‐term toxicity in mice (Figure S2). Together, these findings demonstrate that H_2_S not only enhances motor function recovery following SCI but also exhibits a favorable safety profile, highlighting its promising potential for clinical application.

## 4 Discussion

This study provides compelling evidence for the multifaceted neuroprotective effects of H_2_S in SCI repair. Our data demonstrate that H_2_S effectively remodels the injury microenvironment, thereby creating favorable conditions for motor function recovery. Specifically, H_2_S not only significantly inhibits scar formation, promotes local angiogenesis and the survival of residual neurons and axons, but also suppresses the excessive proliferation and activation of microglia, driving them toward an anti‐inflammatory, reparative phenotype that alleviates neuroinflammation. These findings largely align with previous reports and further support the critical role of H_2_S in reducing secondary injury, promoting tissue repair, and supporting neural function recovery.

Current studies on H_2_S in SCI remain relatively limited, and its neuroprotective mechanisms are not yet fully elucidated. Previous research has utilized H_2_S‐releasing nonsteroidal anti‐inflammatory drugs (e.g., ATB‐346) to concurrently inhibit cyclooxygenase and release H_2_S, which significantly mitigates the inflammatory response and secondary tissue damage following SCI, thereby accelerating motor function recovery [48]. Another study employed a rapidly releasing donor (e.g., NaHS) to enhance the integrity of the blood–spinal cord barrier, reduce endoplasmic reticulum stress and autophagy, and promote functional recovery [28]. Building on these findings, our study further explored the regulatory effects of H_2_S on microglia and demonstrated that H_2_S promotes their polarization toward an anti‐inflammatory, reparative phenotype, thereby revealing a novel mechanism of immune modulation and microenvironment remodeling. In addition, we observed that H_2_S facilitates angiogenesis, further supporting its protective effect on the blood–spinal cord barrier. Recently, research utilizing the slow‐releasing H_2_S donor GYY4137 proposed that H_2_S modulates PANoptosis to inhibit neuronal death, offering novel molecular targets and strategies for treating SCI and related ischemia/reperfusion injuries [29]. Our study further substantiates the neuroprotective effects of H_2_S on neurons and neural fibers in SCI. Notably, after ADT treatment, the number of neurons and neural fibers adjacent to the injury site increased significantly, which may contribute to functional recovery. Recent studies combining H_2_S with biomaterials such as hydrogels have further confirmed the potential therapeutic role of H_2_S in SCI. Local delivery of H_2_S, in conjunction with the specific biological properties of biomaterials, can effectively ameliorate the post‐SCI inflammatory microenvironment and facilitate neural regeneration and functional recovery [49, 50]. In comparison, although intraperitoneal injection of ADT delivers H_2_S to the SCI site and exerts neuroprotective effects, its efficacy in promoting targeted axonal regeneration requires further optimization.

ADT is widely used in clinical practice for liver protection and has a well‐established safety profile. In recent years, ADT has been repurposed as a slow‐release H_2_S donor capable of continuously and stably releasing H_2_S. Using in vivo fluorescence imaging, we detected H_2_S levels at the SCI site following intraperitoneal injection of ADT and demonstrated that a dose of 20 mg/kg effectively sustains H_2_S release. Our study systematically evaluated the reparative effects of H_2_S on scar inhibition, vascular reconstruction, neuroprotection, and inflammation modulation, thereby providing a comprehensive assessment of its overall improvement of the SCI microenvironment. However, whether these key regulatory effects are interrelated and synergistically remodel the injury microenvironment warrants further investigation.

Following SCI, excessive scar formation is a major factor impeding axonal regeneration and functional recovery. This process involves complex interactions among reactive astrocytes, microglia, pericytes, fibroblasts, and the extracellular matrix. These cells secrete inflammatory mediators and chemokines and engage in direct cell–cell interactions to regulate fibrosis and glial scarring. Although these processes initially protect the injured tissue, they ultimately hinder neural regeneration during the chronic phase [11]. Targeting these cells and their interactions is therefore considered a promising strategy for improving SCI repair. Previous studies have shown that systemic administration of microtubule‐stabilizing and antimitotic drugs can effectively inhibit fibroblast migration, reduce scar formation, and promote axonal regeneration [51, 52], while diminishing pericyte‐mediated scarring has also been shown to benefit sensorimotor function [53]. During these processes, microglia may cooperate with pericytes and fibroblasts through direct or indirect signaling to limit immune cell infiltration while preserving tissue integrity [46]. Our study found that H_2_S treatment promotes the transformation of microglia to an anti‐inflammatory reparative phenotype and increases the secretion of anti‐inflammatory mediators, which may help curb excessive proliferation of fibroblasts and pericytes, thereby reducing scar formation and mitigating secondary neuroinflammation. Future studies should further clarify whether microglia mediate the inhibitory effects of H_2_S on scar formation.

Additionally, SCI is accompanied by disruption of the blood–spinal cord barrier and damage to the local vascular network, leading to ischemia and inflammatory responses [54]. Our data indicate that H_2_S treatment significantly increases the density of CD31^+^ microvessels at the injury site, thereby aiding in the reconstruction of the spinal cord vasculature and improving the local microenvironment to support the survival of residual neurons and axons. Although some studies suggest that microglia and macrophages regulate endogenous angiogenesis via signaling pathways such as SPP1 and IGF [55], further exploration is needed to determine whether the mechanisms by which H_2_S promotes angiogenesis align with those of microglia‐mediated endogenous angiogenesis.

The secondary inflammatory response following SCI is a critical factor that exacerbates neuronal damage and functional deficits. As the primary immune cells in the (CNS), microglia play a pivotal role in regulating neuroinflammation, with their polarization state closely linked to energy metabolism. Resting microglia primarily rely on oxidative phosphorylation, whereas activation shifts the pro‐inflammatory phenotype toward glycolysis, with the anti‐inflammatory phenotype remaining dependent on oxidative phosphorylation. AMP‐activated protein kinase (AMPK), a key regulator of cellular energy balance, is essential for maintaining oxidative phosphorylation. Studies have shown that inhibition of AMPK expression in microglia enhances the mTOR‐HIF‐1α signaling pathway, promoting glycolysis and increasing the secretion of inflammatory mediators [56]. Moreover, previous studies have shown that the H_2_S donor ADT can activate the AMPK signaling pathway, improve autophagic flux, and alleviate cellular damage, thereby promoting the polarization of microglia toward an anti‐inflammatory M2 phenotype and reducing pro‐inflammatory cytokine levels [57, 58]. Recent findings further suggest that H_2_S modulates mitochondrial function, reactive oxygen species production [38], and signaling pathways such as PI3K‐Akt, NF‐κB, and Nrf2 to ameliorate the local inflammatory microenvironment [59, 60]. These findings provide clear directions for further exploration of the specific mechanisms by which H_2_S regulates microglial polarization and the inflammatory microenvironment.

In recent years, advances in single‐cell sequencing technologies have revealed the remarkable heterogeneity of microglia. Studies show that, beyond the conventional M1/M2 paradigm, microglia in the injury microenvironment can be further subdivided into multiple functionally distinct subpopulations [61, 62]. Although our findings indicate that H_2_S treatment promotes the conversion of microglia to an anti‐inflammatory, reparative phenotype, it remains unclear whether this effect involves selective regulation of specific subpopulations. Future studies employing single‐cell RNA sequencing, lineage tracing, and multiplex immunostaining are warranted to elucidate the impact of H_2_S on the phenotypic and functional modulation of microglia during SCI repair and to delineate its regulatory effects on particular subpopulations. Such investigations will further clarify the mechanisms by which H_2_S modulates microglial polarization and remodels the injury microenvironment.

Despite the encouraging outcomes of this study, several limitations remain. The precise molecular mechanisms underlying H_2_S‐mediated SCI repair remain unclear and represent a primary focus for future research. Although our data indicate that intraperitoneal injection of ADT does not induce obvious systemic side effects, differences between this administration route and conventional clinical practices warrant further systematic evaluation of its long‐term effects and potential toxicity, as well as exploration of its applicability to other (CNS) injuries. Moreover, further studies involving a more detailed dose–response analysis are warranted to determine the optimal clinical dosage for maximal therapeutic efficacy. Given the complexity of in vivo metabolism, the bioavailability of H_2_S may be limited, potentially affecting its therapeutic efficacy. Consequently, the release rate and systemic distribution of H_2_S following intraperitoneal ADT administration should be assessed using more precise measurement techniques. Future research may also consider developing more efficient drug delivery systems that combine advanced delivery technologies, stem cell therapy, or neurotrophic factors to achieve multi‐target synergistic repair and further enhance therapeutic outcomes.

## 5 Conclusions

In conclusion, this study demonstrates that H_2_S plays a positive role in inhibiting abnormal cell proliferation, promoting angiogenesis, and preserving neurons and axons, while also elucidating its potential mechanism in facilitating functional recovery through modulation of microglial polarization and remodeling of the injury microenvironment.

## Author Contributions

Yu Wang: conceptualization, methodology, investigation, formal analysis, visualization, data curation, writing – original draft, writing – review and editing. Xinyi Jia: investigation, visualization. Yuqi Zhang: investigation. Haibin Shi: investigation. Yuhui Sun: methodology, writing – review and editing. Yaobo Liu: conceptualization, supervision, writing – review and editing, funding acquisition, project administration, resources.

## Conflicts of Interest

The authors declare no conflicts of interest.

## Supporting information


Appendix S1.

## Data Availability

Data supporting the findings of this study are available from the corresponding author upon reasonable request.
