# Supplementary material for: Hydrogen Sulfide Modulates Microglial Polarization and Remodels the Injury Microenvironment to Promote Functional Recovery After Spinal Cord Injury
Source: CNS Neurosci Ther. 2025 May 14;31(5):e70431. doi: 10.1111/cns.70431 (PMC12076064; doi:10.1111/cns.70431)
Supplement: Supplementary file 1 — Appendix S1. [file CNS-31-e70431-s001.zip › cns70431-sup-0001-Figures.docx]

Supplementary Materials for

**Hydrogen Sulfide Modulates Microglial Polarization and Remodels the Injury Microenvironment to Promote Functional Recovery after Spinal Cord Injury**

Yu Wang ^1^, Xinyi Jia ^1^, Yuqi Zhang ^2^, Haibin Shi ^2^, Yuhui Sun ^1^, Yaobo Liu ^1,3, *^

^1^ Jiangsu Key Laboratory of Neuropsychiatric Diseases and Institute of Neuroscience, Soochow University; Clinical Research Center of Neurological Disease, The Second Affiliated Hospital of Soochow University, Suzhou 215123, China.

^2^ State Key Laboratory of Radiation Medicine and Protection, School of Radiation Medicine and Protection, and Collaborative Innovation Center of Radiological Medicine of Jiangsu Higher Education Institutions, Soochow University, Suzhou 215123, P. R. China.

^3^ Co-innovation Center of Neuroregeneration, Nantong University, Nantong 226001, China.

^*^ Corresponding author. E-mail address: liuyaobo@suda.edu.cn (Y. L)

**Supplemental Methods**

**Hematoxylin-eosin (HE) staining**

Tissue sections of the heart, liver, spleen, lung, and kidney from the mouse were prepared by cryosectioning at a thickness of 18 μm. The sections were incubated in hematoxylin for 5 minutes at room temperature. After incubation, the sections were washed with tap water until the tissue exhibited a faint purple hue, ensuring that direct water contact with the tissue was avoided to prevent detachment. The slides were then placed in a slide box containing water at 45°C, and once the tissue on the slide turned blue, they were removed. Following this, the sections were incubated in eosin for 1 minute at room temperature, after which they were washed with tap water to remove excess eosin. Dehydration was performed in 75% alcohol for 10 seconds, followed by 95% alcohol for 10 seconds, and finally 100% alcohol for 30 seconds, all at room temperature. To remove water droplets, the slides were dried by either placing them in an oven set to 65°C or using a blow dryer. The slides were then mounted with neutral balsam and examined under a microscope, where images were captured for further analysis.

**Supplemental Figures**


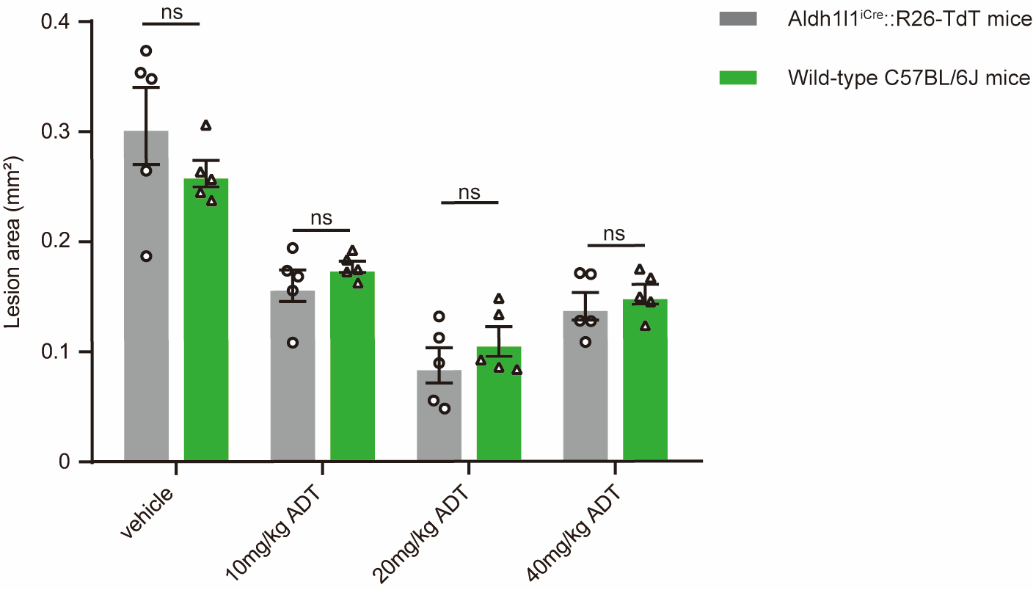


**Fig. S1.** Comparison of lesion areas between Aldh1l1^iCre^::R26-TdT and wild-type C57BL/6J mice across the vehicle, 10, 20, and 40 mg/kg ADT groups. Data are presented as mean ± SEM; n = 5 per group. Statistical analyses were performed using two-way ANOVA followed by Bonferroni’s post hoc test for multiple comparisons. Ns indicates no statistically significant difference.


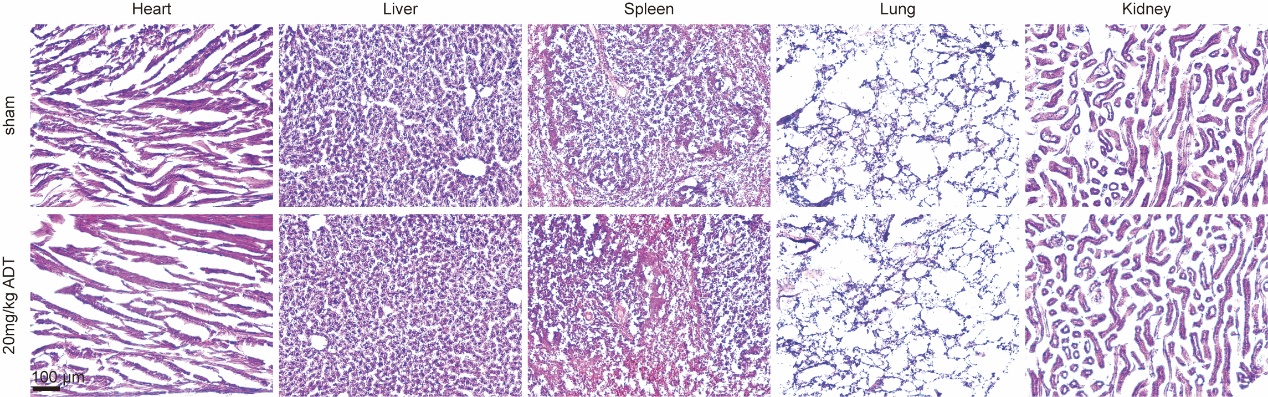


**Fig. S2.** H&E staining of the main organs of mice in the sham and 20mg/kg ADT groups.
