# Supplementary figures and images for: Hydrogen Sulfide Modulates Microglial Polarization and Remodels the Injury Microenvironment to Promote Functional Recovery After Spinal Cord Injury
Source: CNS Neurosci Ther. 2025 May 14;31(5):e70431. doi: 10.1111/cns.70431 (PMC12076064; doi:10.1111/cns.70431)

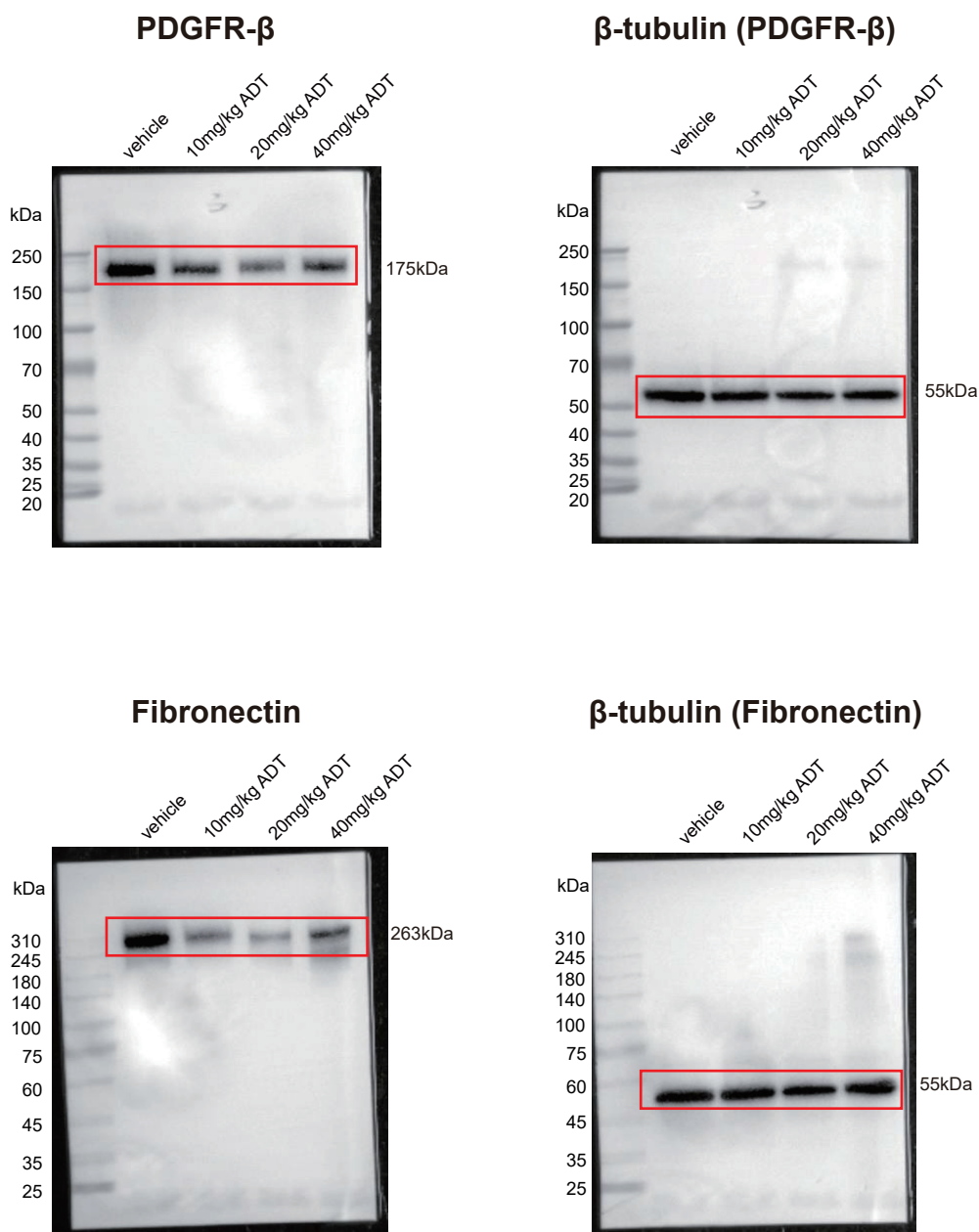

Full unedited blot for Figure 5H

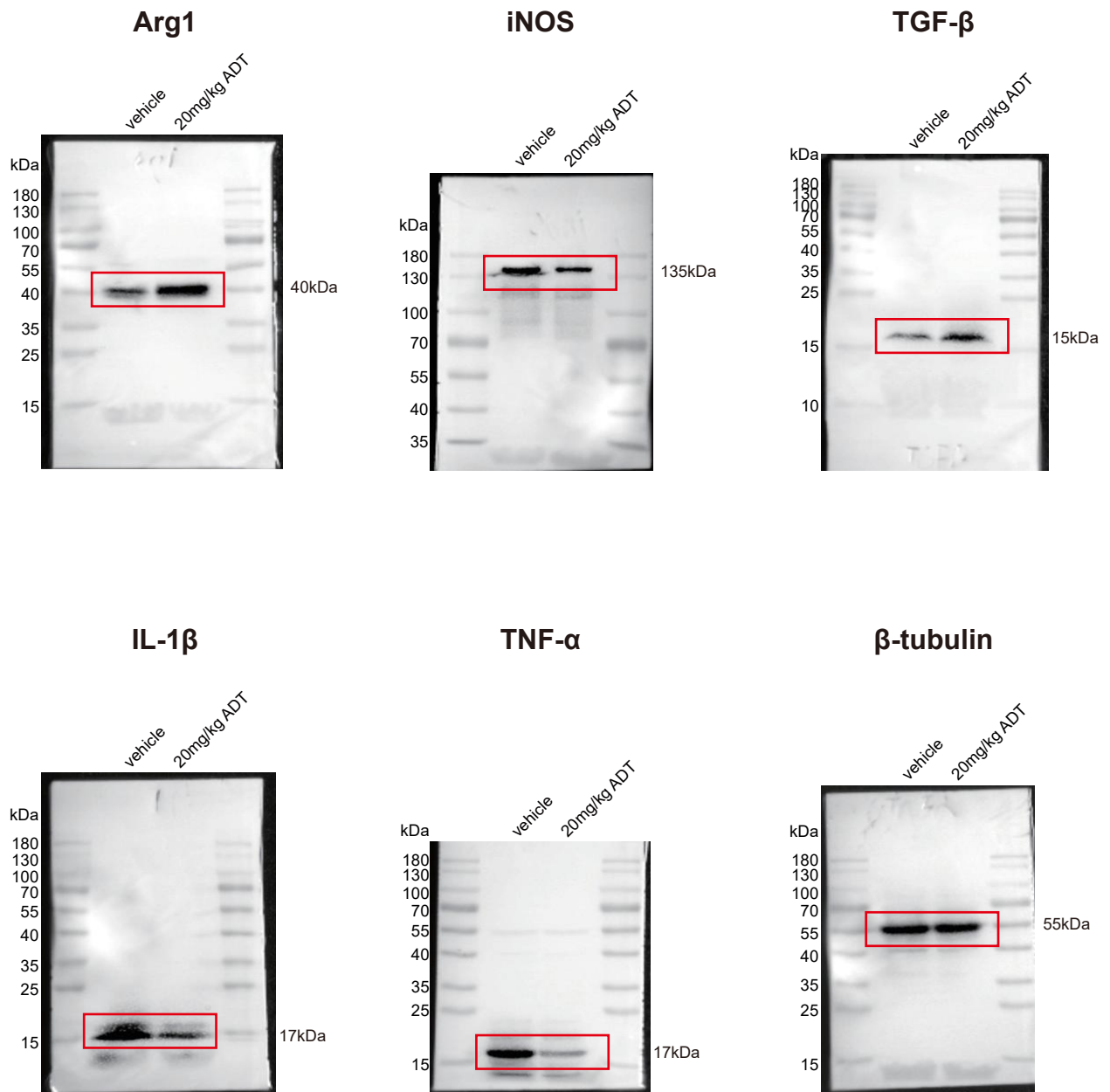

Supplement: Supplementary file 1 — Appendix S1. [file CNS-31-e70431-s001.zip › cns70431-sup-0002-Supinfo.pdf]
